# Preoperative albumin can predict the risk of postoperative deep venous thrombosis in non-cardiac surgery

**DOI:** 10.3389/fmed.2025.1635218

**Published:** 2025-08-21

**Authors:** Shiyan Zhang, Qianyun Pang, Wenjun Liu, Zhu Chen, Ying Wang, Yongting Duan, Hongliang Liu

**Affiliations:** ^1^Department of Anesthesiology, Wansheng Economic and Technological Development Zone People’s Hospital, Chongqing, China; ^2^Department of Anesthesiology, Chongqing University Cancer Hospital, Chongqing, China; ^3^Department of Anesthesiology, Zhuhai City People’s Hospital, The Affiliated Hospital of Beijing Institute of Technology, Zhuhai Clinical Medical College of Jinan University, Zhuhai, Guangdong, China

**Keywords:** serum albumin, deep venous thrombosis, prediction, non-cardiac surgery, intermediate-risk

## Abstract

**Background:**

Postoperative deep venous thrombosis (DVT) is a critical complication of non-cardiac surgery. Hypoalbuminemia reflects both nutritional depletion and inflammation, which may contribute to DVT pathogenesis. In this study, we evaluated preoperative albumin’s association with DVT in patients undergoing elective non-cardiac surgery, and identified risk-stratifying thresholds.

**Methods:**

A retrospective cohort study was conducted involving 2,026 adult patients (exclude local anesthesia cases) undergoing elective non-cardiac surgeries between December 1, 2023, and December 30, 2024. All patients received standardized postoperative surveillance by bilateral lower limb Doppler ultrasound during hospitalization. The primary exposure was preoperative serum albumin level, the primary outcome was postoperative DVT. Multivariable logistic regression was used to analyze the independent risk factors for postoperative DVT, and assess the prediction of preoperative albumin level.

**Results:**

Multivariable logistic regression revealed five independent risk factors for postoperative DVT (preoperative albumin, age, gender, surgical duration, and Caprini score). A linear dose-response relationship was observed between preoperative albumin levels and postoperative DVT incidence from a linear logistic regression. Each 1 g/L decrement in preoperative albumin level increased the risk of postoperative DVT by 8.8% (adjusted OR (aOR): 1.088, 95%CI: 1.028–1.152) when analyzed as a continuous variable. The optimal preoperative albumin cut-off value was 41.9 g/L to predict the risk of postoperative DVT (aOR:2.169, 95% CI:1.144–4.115), and the AUC was 0.885.

**Conclusion:**

Preoperative albumin (the cutoff is 41.9 g/L) may help stratify DVT risk in intermediate-risk non-cardiac surgical patients, though prospective validation is needed given study limitations.

## 1 Introduction

Postoperative deep venous thrombosis (DVT) is a critical complication following non-cardiac surgery, with an incidence ranging from 3.5 to 16% depending on surgical type and patient risk profiles (1–3). It remains a leading cause of prolonged hospitalization, pulmonary embolism, and mortality. Current risk stratification tools, such as the Caprini score, primarily rely on clinical variables and lack integration with objective biomarkers, limiting their precision.

Albumin directly regulates coagulation homeostasis by suppressing platelet activation and fibrin polymerization at physiological levels (4–6). It was reported that low levels of albumin induced experimentally verified hypercoagulability in animal studies (7). Thus, there might be a direct causal link between low level of albumin and the thrombotic risk.

Multiple biomarkers, especially D-dimer, are reported to be related to thrombotic risk, while D-dimer is merely a downstream fibrin degradation product reflecting clot turnover, and can easily be elevated by inflammation/tissue injury before surgery (8). Clinical studies revealed conflicting results regarding the role of preoperative D-dimer in the prediction of postoperative DVT (9–14), while preoperative hypoalbuminemia was an independent risk factor for postoperative DVT in orthopedic, colorectal, and neurosurgical patients (9–12). Albumin levels are stable and routinely measured before surgery, which makes albumin uniquely suited for preoperative risk stratification, as it reflects a patient’s baseline physiological state rather than acute perturbations. But the threshold of preoperative albumin to predict postoperative DVT is still unclear, which makes it difficult to prevent postoperative DVT precisely by regulating preoperative albumin level.

Thus, in this retrospective cohort study, we aimed to characterize the relationship (linear vs. non-linear) between preoperative albumin levels and postoperative DVT in patients undergoing elective non-cardiac surgery; identify critical albumin thresholds for stratifying postoperative DVT risk.

## 2 Materials and methods

### 2.1 Study design

This retrospective cohort study was conducted at Wansheng Economical and Technological Development Zone people’s Hospital, Chongqing, China. The protocol was approved by the Institutional Ethics Committee (Approval number: YYLS2024-153). Informed consent was waived due to the retrospective design and anonymization of patient data. This study adhered to the STROBE (Strengthening the Reporting of Observational Studies in Epidemiology) guidelines, with a completed checklist provided in Supplementary Table 1.

### 2.2 Participant selection

Patients undergoing elective non-cardiac surgery between December 1, 2023, and December 30, 2024 were recruited. The inclusion Criteria included: (1) adults (age ≥ 18 years), (2) under general or regional anesthesia (epidural/spinal/peripheral nerve block), (3) availability of complete records for: preoperative serum albumin levels (measured within 7 days before surgery) or preoperative and postoperative bilateral lower limb vascular ultrasound. The exclusion criteria included: (1) preoperative diagnosis of DVT, (2) emergency surgery or procedures performed under local infiltration anesthesia, (3) missing preoperative albumin levels or incomplete pre-/postoperative vascular ultrasound data, (4) patients undergoing multiple non-cardiac surgeries during the study period (only the first surgery was included).

### 2.3 Data collection

We extracted the following variables from surgical patients who met the inclusion criteria from the electronic medical records: the basic characteristics including demographics [age, sex, body mass index (BMI), clinical status [ASA class, New York Heart Association Functional Class (NYHA), smoking status], the comorbidities [coronary artery disease (CAD), hypertension, diabetes, stroke, asthma, chronic obstructive pulmonary disease (COPD), liver disease, renal disease]; preoperative laboratory parameters including hematology [hemoglobin (Hb), white blood cell count (WBC), platelet count] and biochemistry (albumin, fasting glucose, D-Dimer, creatinine);preoperative Caprini score (retrospectively collected); intraoperative variables including anesthesia methods (general/regional), fluid infusion rate, and surgical duration, bleeding and transfusion.

### 2.4 Primary exposure

Preoperative serum albumin levels were measured in the hospital’s central laboratory using standardized bromocresol green assays. Measurements were obtained after hospital admission but prior to surgery. For patients with multiple preoperative albumin tests, the value closest to the surgical time was selected.

### 2.5 Primary outcome

Postoperative DVT was diagnosed using bilateral lower limb Doppler ultrasound conducted by board-certified radiologists during hospitalization, typically performed 3–7 days post-surgery. DVT cases were identified using ICD-10 codes (I80.207 and I80.209). To minimize misclassification, all DVT diagnoses required confirmation of new-onset thrombosis by comparing preoperative and postoperative Doppler ultrasound reports, asymptomatic DVT detected incidentally on routine screening was included.

### 2.6 Statistical analysis

The basal characteristics of all participants, and DVT cohort or non-DVT cohort were presented as median (interquartile range) for continuous variables, for which, normal distributed variables were compared using Student’s *t*-test, while non-normal distributed variables were compared using Mann-Whitney U test; and presented as frequency (%) for categorical variables, and compared using Fisher exact test. The distributional characteristics of continuous variables were evaluated using the Shapiro-Wilk normality test (*P* ≥ 0.05). Variance homogeneity was verified using Levene’s test (for robustness to non-normality) or Bartlett’s test (when normality assumptions were met).

A linear logistic regression or restricted cubic spline analysis (RCS) was conducted to determine the association between preoperative albumin level and postoperative DVT. The RCS analysis incorporated three knots positioned at the 25th, 50th, and 75th percentiles to capture potential non-linear relationships. The Box-Tidwell method was used to test the linear relationship between the continuous variable and the logit of DVT. Variance inflation factors (VIFs) were calculated for all covariates to address multicollinearity. The adjusted odds ratios (aORs) and 95% confidence intervals (CI) per 1 g/L increment in preoperative albumin level for the risk of postoperative DVT were calculated by multivariate logistic regression when preoperative albumin level was treated as a continuous variable, and the confounding variables for the adjustment were: age, sex, ASA, D-dimer, preoperative Caprini score, infusion rate, surgical type, surgical duration, and anesthesia methods. The cut-off value was determined using Youden’s index which maximizes the sum of sensitivity and specificity. To avoid overfitting, we employ the 10-fold cross-validation method to assess the accuracy and discrimination of the multivariable logistic regression model using the area under the receiver operating characteristic curve (AUC) and Brier scores. Subgroup analyses were stratified by age (<65 years, ≥65 years), sex (male, female), surgical duration (<2 h, ≥2 h), and risk stratification by Caprini score (1-2, 3), and adjusted for the confounding variables mentioned above.

Missing data for preoperative albumin, preoperative lower limb vascular ultrasonic, postoperative DVT diagnosis or other categorical variables led to exclusion of incomplete cases. For continuous variables exhibiting less than 5% missing data, we employed multiple imputation (10 iterations) to handle the missing values. Independent risk factors for postoperative DVT were identified through multivariate logistic regression, and predictive models were evaluated using ROC-derived AUC (incorporating preoperative albumin as continuous or dichotomized variables).

As one individual with preoperative hypoalbuminemia might have a higher D-dimer level (>0.5 mg/L), the interaction effects between albumin (below the cut-off value) and D-dimer (>0.5 mg/L) were also tested to address the potential impact of preoperative D-dimer elevation. A *post hoc* sensitivity analysis excluded patients with extreme preoperative albumin levels (<20 g/L or >50 g/L) to assess the robustness.

Data analyses were conducted using Stata16 (Stata Corp LP, USA). All tests were two-sided, with a *P*-value < 0.05 deemed statistically significant.

## 3 Results

**FIGURE 1:**
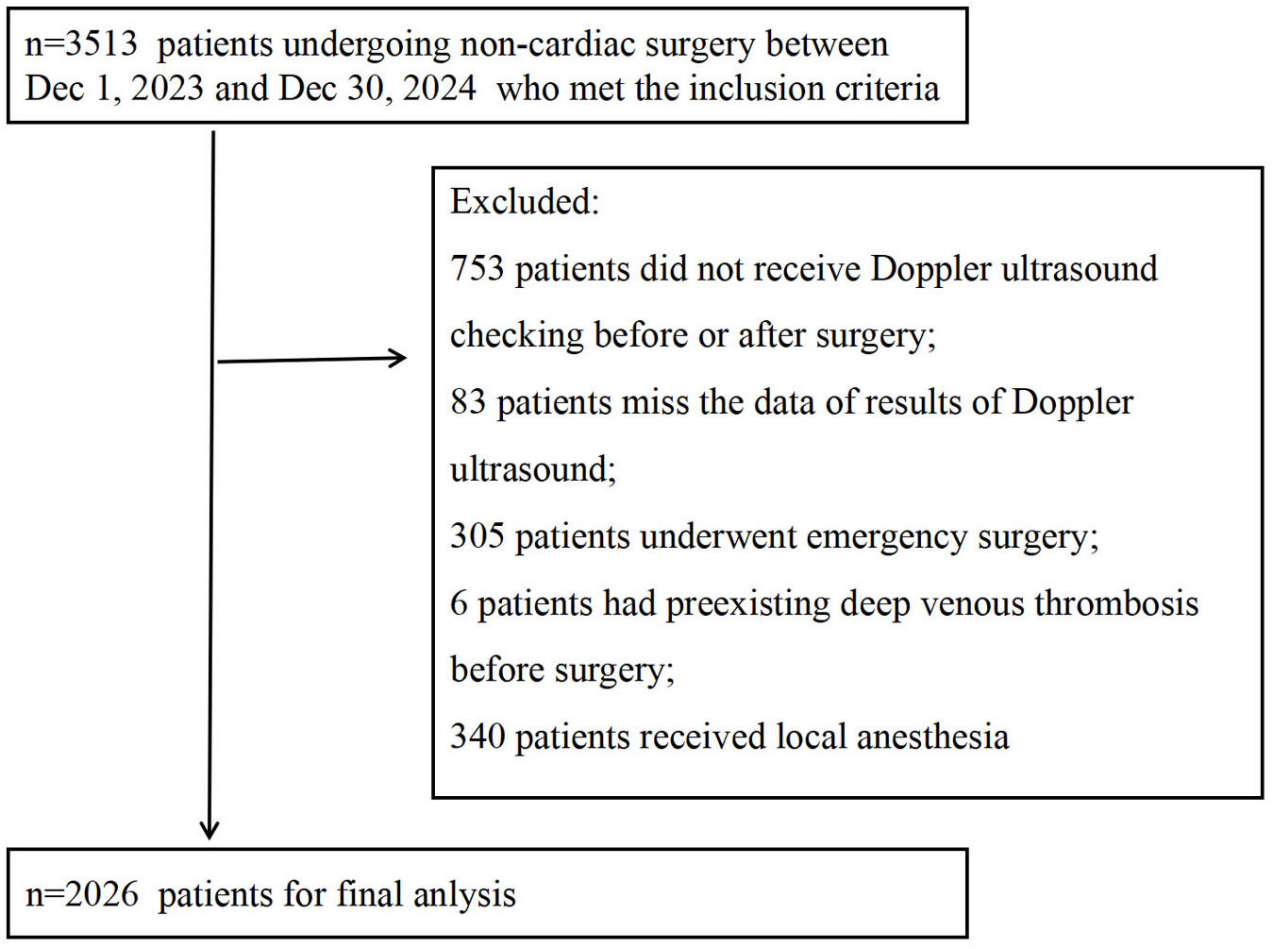
Study flow diagram.

A total of 3513 patients met the inclusion criteria, among these, 753 patients did not receive Doppler ultrasound examination before or after surgery; 83 patients lacked complete Doppler ultrasound results in their medical records although undergoing ultrasound examination; 305 patients underwent emergency surgery; 6 patients had preexisting DVT; and 340 patients received local anesthesia (administered by surgeon). After these exclusions, 2026 patients were included in the final statistical analysis (Figure 1). All patients had a Caprini score ≤3 and received mechanical prophylaxis. Postoperative DVT occurred in 3.55% (*n* = 72) of the cohort. The basic characteristics in all participants, DVT cohort, and non-DVT cohort were summarized in Table 1.

**TABLE 1:** The basic characteristics in all participants, DVT cohort, and non-DVT cohort.

Variables	Total (*n* = 2026)	Non-DVT (*n* = 1954)	DVT (*n* = 72)	Statistic	*P*
Age(y), M (Q_1_, Q_3_)	55 (44, 67)	54 (44, 67)	70 (64.8, 78.3)	*Z* = −8.42	<0.001
Preoperative D2, M (Q_1_, Q_3_)	0.34 (0.11, 1.29)	0.24 (0.10, 1.29)	1.29 (0.54, 3.16)	*Z* = −13.83	<0.001
Postoperative D2, M (Q_1_, Q_3_)	4.47 (0.98, 4.47)	4.47 (0.97, 4.47)	3.60 (1.08, 4.47)	*Z* = −0.44	0.660
BMI(kg/cm^2^), M (Q_1_, Q_3_)	24.09 (21.84, 26.56)	24.09 (21.88, 26.57)	23.84 (21.48, 25.97)	*Z* = −1.00	0.319
Hb(g/L), M (Q_1_, Q_3_)	132 (121, 145)	134 (123, 146)	120 (106, 134)	*Z* = −10.70	<0.001
ALB(g/L), M (Q_1_, Q_3_)	43.6 (40.3, 46.3)	43.7 (40.7, 46.4)	38.4 (34.3, 42.0)	*Z* = −7.96	<0.001
Glucose(mmol/L), M (Q_1_, Q_3_)	6.1 (5.3, 6.8)	6.1 (5.3, 6.8)	6.1 (5.2, 7.4)	*Z* = −0.262	0.794
Creatinine, M (Q_1_, Q_3_)	66 (55, 78)	66 (55, 78)	67 (54, 79)	*Z* = −0.10	0.921
PLT(×10^9^/L), M (Q_1_, Q_3_)	229 (190, 276)	230 (192, 274)	222 (172, 284)	*Z* = 1.76	0.079
WBC(×10^9^/L), M (Q_1_, Q_3_)	7.02 (5.61, 8.97)	6.90 (5.50, 8.76)	7.74 (6.25, 9.91)	*Z* = −5.08	<0.001
Surgicalduration(min), M (Q_1_, Q_3_)	70 (40, 115)	70 (40, 115)	141.5 (95, 221.3)	*Z* = −8.18	<0.001
Infusion Rate(ml/kg/h), M (Q_1_, Q_3_)	9.0 (6., 12.9)	9.1 (6.7, 13.0)	8.2 (5.9, 10.1)	*Z* = −2.91	0.004
Bleeding(ml), M (Q_1_, Q_3_)	20 (5, 100)	20 (5, 100)	100 (50, 200)	*Z* = −6.18	<0.001
Gender, *n* (%)				χ^2^ = 4.82	0.028
Female	1152 (56.86)	1102 (56.40)	50 (69.44)		
Male	874 (43.14)	852 (43.60)	22 (30.56)		
ASA, *n* (%)				χ^2^ = 50.53	<0.001
ASA ≤ 2	1327 (65.50)	1308 (66.94)	19 (26.39)		
ASA > 2	699 (34.50)	646 (33.06)	53 (73.61)		
NYHA, *n* (%)				–	0.183
NYHA ≤ 2	2004 (98.91)	1934 (98.98)	70 (97.22)		
NYHA > 2	22 (1.09)	20 (1.02)	2 (2.78)		
Anesthesia method, *n* (%)				χ^2^ = 32.06	<0.001
GA	1310 (64.66)	1286 (65.81)	24 (33.33)		
GA + LA	716 (35.34)	668 (34.19)	48 (66.67)		
Smoking, *n* (%)				χ^2^ = 2.35	0.125
No	1663 (82.08)	1599 (81.83)	64 (88.89)		
Yes	363 (17.92)	355 (18.17)	8 (11.11)		
Hypertension, *n* (%)				χ^2^ = 3.66	0.056
No	1492 (73.64)	1446 (74.00)	46 (63.89)		
Yes	534 (26.36)	508 (26.00)	26 (36.11)		
Diabetes, *n* (%)				χ^2^ = 1.17	0.280
No	1821 (89.88)	1759 (90.02)	62 (86.11)		
Yes	205 (10.12)	195 (9.98)	10 (13.89)		
Coronary artery disease, *n* (%)				χ^2^ = 4.20	0.040
No	1902 (93.88)	1839 (94.11)	63 (87.50)		
Yes	124 (6.12)	115 (5.89)	9 (12.50)		
Stroke, *n* (%)				χ^2^ = 16.15	<0.001
No	1901 (93.83)	1842 (94.27)	59 (81.94)		
Yes	125 (6.17)	112 (5.73)	13 (18.06)		
VTErisk stratification, *n* (%)				χ^2^ = 82.29	<0.001
Low	1733 (85.54)	1698 (86.90)	35 (48.61)		
Median	293 (14.46)	256 (13.10)	37 (51.39)		
Surgical type, *n* (%)				χ^2^ = 50.81	<0.001*
General surgery	498 (24.58)	485 (24.82)	13 (18.06)		
Gynaecology and obstetrics surgery	358 (17.67)	354 (18.12)	4 (5.56)		
Urological surgery	430 (21.22)	428 (21.90)	2 (2.78)		
Orthopedic surgery	531 (26.21)	489 (25.03)	42 (58.33)		
Head surgery	161 (7.95)	153 (7.83)	8 (11.11)		
others	48 (2.37)	45 (2.30)	8 (11.11)		

Z, Mann-Whitney test; χ^2^, Chi-square test, –, Fisher exact; *, Simulated *p*-value; M, Median; Q_1_, 1st Quartile; Q_3_, 3st Quartile; ASA, American Society of Anesthesiologists classification score; ALB, albumin; BMI, body mass index; Hb, hemoglobin, LA, local anesthesia technique; GA, general anesthesia; NYHA, New York Heart Association classification; PLT, platelet; VTE, venous thrombosis; WBC, white blood cell.

**FIGURE 2:**
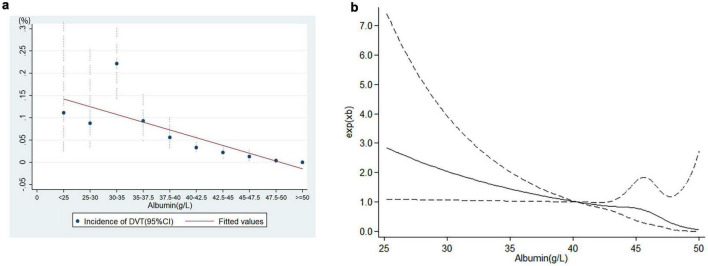
The relationship between albumin level and deep venous thrombosis. **(a)** Linear relationship, **(b)** restricted cubic spline analysis (RCS). A linear dose-response relationship exists between preoperative albumin levels and postoperative DVT risk.

**FIGURE 3:**
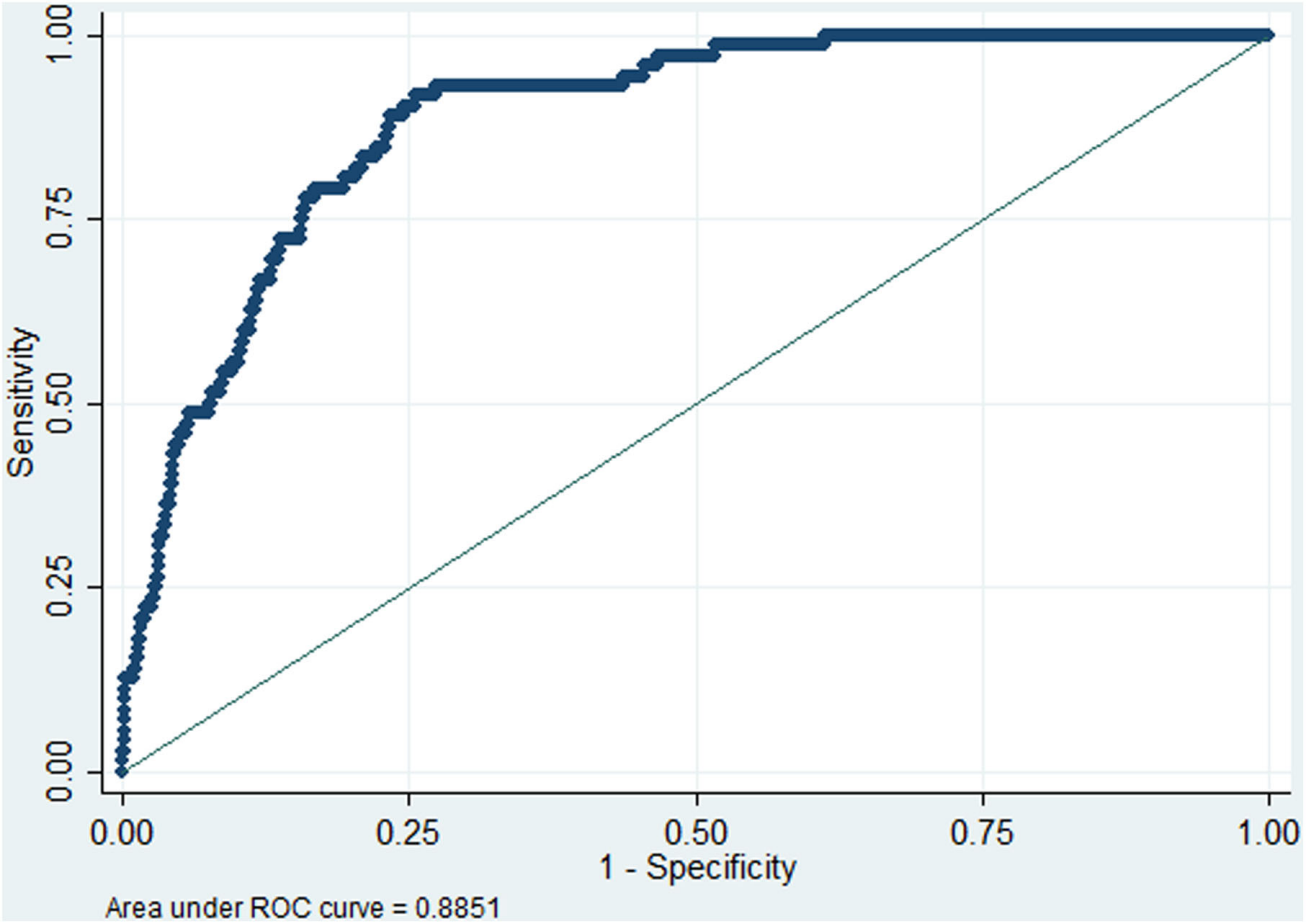
Receiver operating characteristic (ROC) curve analysis of preoperative albumin (dichotomized at 41.9 g/L) for predicting the risk of postoperative deep venous thrombosis. The area under Receiver operating characteristic curve (AUC) was 0.885, indicating high discrimination.

Linear logistic regression demonstrated a significant dose-response relationship between preoperative albumin levels and postoperative DVT incidence (Figure 2a), and RCS analysis confirmed the linearity of this association (Figure 2b), and *P* = 0.098 for non-linearity test via Box-Tidwell procedure. Each 1 g/L decrement in preoperative albumin level increased the risk of postoperative DVT by 8.8% (adjusted OR (aOR): 1.088, 95%CI: 1.028–1.152) when analyzed as a continuous variable. The optimal preoperative albumin cut-off value was 41.9 g/L to predict the risk of postoperative DVT (aOR:2.169, 95%CI:1.144–4.115), and the AUC was 0.885 (Figure 3). Five independent risk factors were identified for postoperative DVT from multivariate logistic regression: preoperative albumin, age, gender, surgical duration, and Caprini score (VTE risk stratification) (VIF was between1.160 and 1.576) (Table 2). The AUC was 0.885–0.890 (incorporating preoperative albumin as a categorized or a continuous variable). The10-fold cross internal validation results showed that the AUC was 0.840–0.933, the Brier score was 0.012–0.056 (Supplementary Table 2).

**TABLE 2:** Univariate and multivariate logistic regression analysis.

Variables	Univariate analysis		Multivariate analysis	
	OR (95%CI)	*P*	aOR (95%CI)	*P*
Age(y)	1.079 (1.059, 1.100)	0.000	1.063 (1.034, 1.092)	0.000
Gender (male vs. female)	0.569 (0.342, 0.947)	0.030	0.484 (0.270, 0.868)	0.015
ASA > 2 vs. ASA ≤ 2	5.648 (3.316, 9.620)	0.000	0.878 (0.432, 1.786)	0.720
NYHA > 2 vs. NYHA ≤ 2	2.762 (0.633, 12.052)	0.176		
BMI(kg/cm^2^)	0.959 (0.896, 1.027)	0.233		
Smoking (yes vs. no)	0.563 (0.268, 1.185)	0.130		
Preoperative D-dimer (mg/L)	1.262 (1.163, 1.369)	0.000	1.008 (0.901, 1.127)	0.891
Postoperative D-dimer (mg/L)	1.071 (0.953, 1.230)	0.251		
ALB(g/L)	0.853 (0.821, 0.886)	0.000	0.914 (0.864, 0.966)	0.001
PLT(×10^9^/L)	0.999 (0.995, 1.002)	0.436		
Glucose(mmonl/L)	0.973 (0.856, 1.107)	0.679		
WBC(×10^9^/L)	1.053 (1.000, 1.109)	0.051		
VTErisk stratification (median vs. low)	7.011 (4.337, 11.337)	0.000	1.847 (1.010, 3.378)	0.046
Anesthesia method (GA + LA vs. GA)	3.850 (2.338, 6.341)	0.000	1.805 (0.873, 3.730)	0.111
Surgicalduration (min)	1.011 (1.008, 1.013)	0.000	1.010 (1.006, 1.014)	0.000
**Surgical type**				
General surgery	Reference			
Gynaecology and obstetrics surgery	0.422 (0.136,1.303)	0.134	0.670 (0.186,2.409)	0.539
Urological surgery	0.174 (0.039,0.777)	0.022	0.462 (0.097,2.208)	0.334
Orthopedic surgery	3.204 (1.699,6.044)	0.000	0.571 (0.261,1.249)	0.161
Head surgery	1.951 (0.794,9.054)	0.145	1.642 (0.530,5.080)	0.390
Others	2.487 (0.683,9.054)	0.167	0.735 (0.157,3.433)	0.695
Bleeding (ml)	1.001 (1.001,1.002)	0.000	1.000 (0.999,1.001)	0.817
Infusion Rate (ml/kg/h)	0.933 (0.886,0.983)	0.009	1.001 (0.949,1.056)	0.976

OR, odds ratio; CI, confidence interval; aOR, adjusted odds ratio; ASA, American Society of Anesthesiologists classification score; ALB, albumin; BMI, body mass index; GA, general anesthesia; LA, local anesthesia technique; NYHA, New York Heart Association classification; PLT, platelet; WBC, white blood cell.

Subgroup analysis revealed that elderly male patients, underwent general anesthesia combined with local anesthesia, or surgical duration >2 h were more likely to develop postoperative DVT when preoperative albumin level was below the cut-off value (Table 3).

**TABLE 3:** Subgroup analysis of the prediction below cutoff value.

Variable	Subgroup	aOR, 95%CI	*P*-value
Age	>65 y ≤65 y	3.072 (1.298, 7.266) Reference	0.011
Gender	Male Female	0.453 (0.230, 0.895) Reference	0.023
Anesthesia method	GA + LA GA	4.525 (1.626, 12.592) Reference	0.004
Surgical duration	≥2 h <2 h	2.059 (1.013, 4.182) Reference	0.046
D-dimer	≥0.5 <0.5	2.163 (0.885, 5.290) Reference	0.091

aOR, adjusted odds ratio; CI, confidence interval; GA, general anesthesia; LA, local anesthesia technique.

Interaction analysis demonstrated no significant effect modification between preoperative albumin (below 41.9 g/L) and D-dimer (>0.5 mg/L) (*P* = 0.935).

In *post hoc* sensitivity analysis, patients with extreme preoperative albumin levels (<20 g/L or >50 g/L) were excluded. Each 1 g/L decrement of preoperative albumin level increased DVT risk by 8.9% (aOR:1.089, 95%CI: 1.029–1.152). Preoperative albumin below the cut-off value predicted postoperative DVT with an aOR of 2.083 (95%CI: 1.100–3.937).

## 4 Discussion

In this study, preoperative albumin levels demonstrated a linear correlation with postoperative DVT risk in non-cardiac surgery patients, a preoperative albumin level < 41.9 g/L served as a critical threshold to predict postoperative DVT.

Physiologically, albumin within normal range inhibits platelet aggregation and enhances neutralization of coagulation factor Xa, thereby exerting anticoagulation effects (4, 15). An *in vitro* study showed that hypoalbuminemia promoted primary hemostasis, accelerates platelet aggregation, and strengthens clot formation (7), suggesting a potential mechanism for hypercoagulability. This aligns with clinical observations linking hypoalbuminemia to elevated DVT risk and mortality in cancer patients (16, 17). Several clinical studies identified preoperative hypoalbuminemia as an independent risk factor for postoperative DVT (9–12), but conflicting results exist (13). In which, elderly patients underwent hip arthroplasty, and the preoperative albumin levels were low in both groups (DVT and non-DVT groups), so the role of preoperative albumin in DVT may depend on patients, surgical type, and preoperative nutrition status.

Our findings confirmed that preoperative albumin was an independent predictor of postoperative DVT in non-cardiac surgery, with a high AUC value of 0.778 to predict postoperative DVT when below 41.9 g/L; and with high values of AUC when combined with the other 4 independent risk factors (0.885–0.890 as a categorized or a continuous variable), internal validation results also supporting its potential as a biomarker for DVT risk assessment.

Contrary to reports of a non-linear relationship between albumin and preoperative DVT in geriatric patients with hip fractures (18), our study exhibited a linear dose-response relationship. Each 1 g/L increase in preoperative albumin reduced postoperative DVT risk by 8.8%, the cut-off value was 41.9 g/L, underscoring the clinical relevance of correcting preoperative hypoalbuminemia as a targeted preventive strategy.

The role of D-dimer remains contentious; some studies have identified it as an independent predictor of postoperative DVT (9, 10, 13, 14), while others have not (11, 12). In our cohort, D-dimer was not found to be an independent risk factor, possibly due to differences in patient selection across various research studies. And subgroup analysis also revealed that elderly male patients, patients with surgical duration >2 h, or general anesthesia combined with regional anesthesia were more likely to develop postoperative DVT when preoperative albumin level was below the cut-off value. As aging could increase blood viscosity and endothelial dysfunction, elevate clotting factors, and reduce antithrombin (19). The interaction analysis in our study revealed no statistically significant effect modification between preoperative albumin levels (dichotomized at < 41.9 g/L) and elevated D-dimer levels (>0.5 mg/L) on postoperative DVT. Their contributions appear to be largely independent. Future studies with larger samples or different analytical approaches could further explore potential synergies.

There are some limitations of this study. First, this is a single-center retrospective study, the results might be affected by geographical location and medical level, and should be cautiously extrapolated to other institutions; second, some other factors such as postoperative nutrition and mobility might influence the postoperative albumin fluctuations, which were not recorded and retrieved, but might confound postoperative DVT risk. Despite these constraints, our findings highlighted preoperative albumin as a predictive biomarker and intervention target. However, the study population did not include individuals at critical thrombotic risk. Consequently, the model and conclusions are not applicable for guiding preventive decisions in extremely high-risk thrombosis patients, who should continue to follow existing intensified prevention guidelines based on the Caprini score. Despite this limitation in broad applicability, this research focuses on a large and clinically challenging group (intermediate-to-low risk patients). Within this cohort, identifying individuals with thrombotic risk exceeding Caprini score predictions (e.g., patients with Caprini score 2 but low albumin) holds significant clinical importance. For such patients, intensification of prophylactic measures may be warranted, though future studies are required to validate this approach.

In conclusion, preoperative albumin (the cutoff is 41.9 g/L) is may help stratify DVT risk in intermediate-risk non-cardiac surgical patients, though prospective validation is needed given study limitations.

## Data Availability

The raw data supporting the conclusions of this article will be made available by the authors, without undue reservation.
