# Supplementary material for: Preoperative albumin can predict the risk of postoperative deep venous thrombosis in non-cardiac surgery
Source: Front Med (Lausanne). 2025 Aug 21;12:1635218. doi: 10.3389/fmed.2025.1635218 (PMC12408617; doi:10.3389/fmed.2025.1635218)
Supplement: Supplementary file 2 [file Table_2.docx]

Supplemental Table 2 The 10-fold cross-internal-validation results

| Model | AUC, 95%CI | *P* value | Brier |
| --- | --- | --- | --- |
| 1 | 0.866 (0.816, 0.958) | 0.000 | 0.038 |
| 2 | 0.933 (0.852, 1.000) | 0.002 | 0.017 |
| 3 | 0.903 (0.835, 0.971) | 0.000 | 0.043 |
| 4 | 0.840 (0.764, 0.917) | 0.000 | 0.056 |
| 5 | 0.904 (0.835, 0.973) | 0.000 | 0.034 |
| 6 | 0.896 (0.850, 0.941) | 0.027 | 0.012 |
| 7 | 0.852 (0.700, 1.000) | 0.001 | 0.024 |
| 8 | 0.922 (0.848, 0.996) | 0.001 | 0.020 |
| 9 | 0.891 (0.808, 0.974) | 0.001 | 0.029 |

AUC: the area under the receiver operating characteristic curve; CI: Confidence Interval.‌
